# Ligand‐complex–based quantification of β2‐integrin–mediated affinity and avidity of murine T cells

**DOI:** 10.1002/ame2.70155

**Published:** 2026-02-25

**Authors:** M. Therre, A. A. Kuhnle, H. M. Arndt, N. Frey, M. H. Konstandin, N. V. Bogert

**Affiliations:** ^1^ Department of Cardiology, University Hospital Heidelberg Ruprecht‐Karls‐University Heidelberg Heidelberg Germany; ^2^ German Centre for Cardiovascular Research (DZHK), Partner Site Heidelberg/Mannheim Berlin Germany; ^3^ Department of Cardiology, Angiology and Intensive Care Medicine Saarland University Hospital Homburg Germany; ^4^ Department of Gynecology and Obstetrics University Hospital Heidelberg Heidelberg Germany

**Keywords:** affinity, avidity, ICAM‐1, T cells, T helper cells, β2‐integrin

## Abstract

**Background:**

Integrins facilitate binding to the extracellular matrix and other cells. Their subunit β2 is exclusively expressed by leukocytes, binds to the intercellular cell adhesion molecule 1 (ICAM‐1), and is pivotal for their recruitment to sites of inflammation such as the atherosclerotic plaque.

**Methods:**

To investigate β2‐integrin–mediated adhesiveness, a well‐established assay for human whole blood was adapted for the analysis of murine T cell subsets. Changes in avidity and affinity were assessed by incubation of murine complexes ICAM‐1 in murine whole blood and consecutive stimulation with PMA and Mg^2+^/EGTA. Underlying signaling pathways in β2‐integrin–mediated adhesiveness upon chemokine stimulation with CCL‐19 were identified by incubation with reducing substances, and a Ca^2+^ chelator and ROS and Ca^2+^ measurements were carried out.

**Results:**

Incubation of murine whole blood with PMA leads to 30‐fold and Mg^2+/^EGTA to 65‐fold increase in β2‐integrin–mediated adhesiveness of T cells. Specificity of the assay was proven by preincubation of a blocking antibody, leading to a 60% reduction in adhesion capacity. ROS species and Ca^2+^ are crucial for chemokine‐mediated β2‐integrin activation. In vivo relevance was proven by induction of T cell adhesiveness in whole blood of mice upon myocardial infarction.

**Conclusions:**

Our assay allows specific quantification of β2‐integrin–mediated affinity and avidity of T cells in whole blood samples. In congruence to human adhesion, these mechanisms are ROS and Ca^2+^ dependent and significantly elevated after myocardial infarction. Our refined and robust assay may be of particular use in phenotyping involved mechanisms in T cell activation in atherosclerotic cardiovascular disease.

## Graphical abstract

Integrin subunit β2 of murine T cells binds to the intercellular cell adhesion molecule 1 (ICAM‐1), which was fluorescently labeled to quantify β2‐integrin activation with fluorescence‐activated cytometry. Integrins remain inactive until they are activated. This activation process is called adhesion and can be induced through changes in receptor avidity or affinity. Changes in avidity and affinity were assessed by incubation of murine complexes ICAM‐1 in murine whole blood and consecutive stimulation with PMA and Mg^2+^. Specificity of the assay was proven by preincubation of a blocking antibody. Inhibitor experiments show that reactive oxygen species (ROS) and calcium (Ca^2+^) are crucial for chemokine (CCL‐17 and its receptor CCR7)‐mediated β2‐integrin activation.

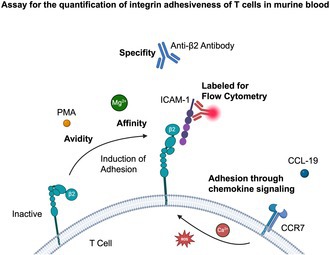

## 1 INTRODUCTION

Leukocyte extravasation classically follows three steps.^1^, ^2^ After being tethered by selectins, leukocytes are activated by chemokines for the initiation of adhesion. Consequently, integrins, as principal adhesion proteins of the cell, firmly adhere to their respective ligands.^3^ Each molecule is heterodimeric and consists of two transmembrane subunits called α and β, resulting in 24 distinctive integrin combinations.^4^ Integrin‐β2 (also known as CD18) together with subunit αL forms the leukocyte‐function–associated‐antigen‐1 (LFA‐1), which binds to the intercellular cell adhesion molecule‐1 (ICAM‐1).^5^

The complex adhesion process is regulated via changes in affinity or avidity of integrin receptors.^6^, ^7^, ^8^, ^9^ Affinity describes the strength of a single binding site by conformational change of the integrin, which can experimentally be induced after binding of divalent cations such as magnesium (Mg^2+^) to the metal ion–dependent adhesion site (MIDAS) of the integrin.^10^ The cumulative binding strength of multivalent integrin interactions is caused by lateral movement and clustering of the receptors and defined as avidity that can be increased, for example, by stimulation with Phorbol‐12‐myristat‐13‐acetat (PMA) or chemokines such as chemokine (C–C motif) ligand 19 (CCL‐19).^9^, ^11^ CCL‐19 binds chemokine (C–C motif) receptor 7 (CCR7), which is expressed by nearly all T cell subsets acting as global T cell adhesion inductor.^12^ Both affinity and avidity can be analyzed through applying the recently established ligand‐complex–based adhesion assay (LC‐AA) in human isolated cells or whole blood.^13^, ^14^ However, up to now, there is no method available that allows quantification of β2‐integrin activation on a single cell level in murine whole blood samples. The newly designed murine ligand‐complex–based adhesion assay (mLC‐AA) offers a fast and simple evaluation of β2‐integrin avidity and affinity in murine T cell subpopulations.

Greater understanding of this process may help identify therapeutic targets in inflammatory diseases such as chronic coronary syndrome (CCS) and myocardial infarction (MI), where the relevance of inflammation in disease progression^15^, ^16^ and the need for specific anti‐inflammatory therapies^16^, ^17^, ^18^ have been recognized. In a previously conducted study, we were able to show that reactive oxygen species (ROS) and calcium (Ca^2+^) signaling is essential for β2‐integrin activation on T cells upon chemokine stimulation in humans.^19^ Interestingly, β2‐integrin activation on T cells is crucial for the recruitment of T cells within atherosclerotic plaques^19^, ^20^ and can be used for risk prediction of cardiovascular events.^21^ The importance of ROS/Ca^2+^ signaling upon chemokine stimulation in murine T cells has not been investigated till now. Application of the mLC‐AA may therefore help characterize in vivo signaling cascades and pathogenetically important subpopulations contributing to CCS or MI.

## 2 MATERIAL AND METHODS

### 2.1 Animals

Experiments comply with the regulatory European standards of Directive 2010/63/EU and are reported according to the ARRIVE guidelines. All conducted animal experiments of this study were reviewed and approved by the Regierungspräsidium Karlsruhe (G246/14; T24/19). Animals were 8‐week‐old male C57BL/6 mice that were primary assigned to investigate other experimental approaches, and blood was collected only postmortal as a by‐product according to the 3R principle. They were housed under standardized conditions with access to standard chow 1320 and drinking water ad libitum. For all experiments, except for MI, randomization to different groups was not necessary and no animals were excluded. Mice were killed by cervical dislocation, and approximately 80–100 μL of blood was aspirated by intracardial punctation before organ removal. Collected blood was transferred in a 1.5 mL Eppendorf tube containing 10 μL of sodium heparin (B. Braun SE).

### 2.2 Myocardial infarction

Twelve 8‐week‐old C57BL/6 mice were randomly assigned to either permanent LAD ligation (MI, *n* = 6) or sham surgery (SHAM, *n* = 6). There were no significant differences between groups based on the body weight. MI was performed by permanent ligation of the left anterior descending (LAD) artery under isoflurane anesthesia and preoperative analgesia with buprenorphine. Sham surgery was identical, however, without LAD occlusion. Postoperative analgesia was applied for the following days. Mice were sacrificed 7 days after induction of MI by cervical dislocation, and blood was collected as previously described.

### 2.3 Antibodies and reagents

Recombinant Mouse ICAM‐1/CD54 Fc chimera protein was purchased from R&D Systems, and Allophycocyanin (APC) AffiniPure F(ab′)2 fragment donkey anti‐human IgG (Fcγ fragment specific) was purchased from Jackson ImmunoResearch. FACS Lyse/Fix solution and the following antibodies were purchased from BD/Pharmingen: CD3‐BV421 (clone 17A2), CD4‐PE (clone H129.19), CD44‐FITC (clone IM7), CD62L‐PE‐Cy™7 (clone MEL‐14), and blocking CD18‐specific IgG (clone GAME) as well as rat IgG1κ isotype control (clone R3–34). Recombinant murine MIP‐3β (CCL19) was purchased from PeproTech. PMA, ethylene glycol tetraacetic acid (EGTA), apocynin (APO), *N*‐acetylcysteine (NAC), BAPTA‐AM (BAPTA), diphenyleneiodonium chloride (DPI), and all other chemicals were purchased from Sigma unless stated otherwise. FACS buffer contained PBS supplemented with 0.5% bovine serum albumin (BSA) and 5% fetal calf serum (FCS).

### 2.4 Ligand‐complex–based adhesion assay and flow cytometry

Flow cytometry–based methods to quantify β2‐integrin–mediated adhesiveness in human purified T cells and whole blood were established to assess avidity‐ as well as affinity‐mediated changes.^13^, ^14^ For analysis of β2‐integrin–mediated adhesiveness in murine whole blood, ICAM‐1–Fc‐F(ab′)2 complexes (scICAM‐1) were generated. Per assay, APC AffiniPure F(ab′)2 fragment donkey anti‐human IgG was diluted 1:10 and ICAM‐1–Fc (200 μg/mL) was diluted 1:3.2 in PBS and incubated in a 1.5 mL Eppendorf tube at 4°C overnight. The next day, 20 μL of freshly sampled murine whole blood was transferred into a FACS tube (Fisher Scientific) and diluted 1:1 in PBS^+^ (PBS containing 0.5% BSA, 2 mmol/L Mg^2+^, and 1 mmol/L Ca^2+^). In all ROS/Ca^2+^ inhibition experiments, blood was preincubated 1:1 with the respective agent in PBS^+^ for 60 min at 37°C, followed by three washing steps with PBS. APO is a methoxy‐substituted and cell‐permeable catechol acting as potent inhibitor of NADPH‐oxidase (NOX). NAC is a precursor to the amino acid cysteine, which is essential for the synthesis of the key antioxidant glutathione and can also inhibit NOX. DPI is a cell‐permeable inhibitor of NOX and nitric oxide synthase. The acetoxymethyl ester of 1,2‐Bis(2‐aminophenoxy)ethane‐tetraacetic acid (BAPTA‐AM) is a cell‐permeable and highly selective Ca^2+^ chelator. Final concentrations of the three reducing agents were 5 mmol/L (APO), 10 mmol/L (NAC), and 20 μmol/L (DPI). Final concentration of the Ca^2+^ chelator BAPTA was 200 μmol/L. For assay initiation, 10 μL prepared scICAM‐1 and 15 μL PBS^+^ containing CCL‐19, PMA or Mg^2+^/EGTA stimulation or PBS^+^ as solvent control was added. When not indicated otherwise, CCL‐19 stimulation (final concentration 40 nmol/L) was performed for 2 min, whereas Mg^2+^/EGTA (10 mmol/L/1 mmol/L) and PMA (300 nmol/L) treatment was performed for 30 min. To terminate the reaction, 650 μL of BD Lyse/Fix, under the same cationic conditions used during stimulation, was added. After 5 min, fixation was stopped by adding ice‐cold FACS buffer, and tubes were transferred onto ice. When not indicated otherwise, CCL‐19 stimulation (final concentration 40 nmol/L) was performed for 2 min, whereas Mg^2+^/EGTA (10 mmol/L/1 mmol/L) and PMA (300 nmol/L) treatment was performed for 30 min. After fixation, cells were pelleted and stained for surface markers as indicated by incubation on ice for 30 min in the dark. Naive T helper (Th) cells were defined as CD44^−^CD62L^+^, effector memory cells as CD44^+^CD62L^−^, and central memory cells as CD44^+^CD62L^+^ as previously described.^22^ Cells were washed once and analyzed on a FACSVerse flow cytometer (BD Biosciences). Experiments were analyzed using FlowJo 10.8.2.

### 2.5 ROS and calcium assay

Spleens were harvested from the killed mice and homogenized by passing the tissue through a 70 μm nylon cell strainer (Greiner Bio‐One). Erythrocytes were lysed using a hypotonic buffer containing 0.15 mol/L ammonium chloride, 1 mmol/L potassium bicarbonate, and 0.1 mmol/L disodium EDTA in distilled water. For the detection of ROS, T cells were incubated with 1 μmol/L ROS Assay Stain Concentrate (Total Reactive Oxygen Species Assay Kit, Thermo Fisher Scientific) for 30 min at 37°C. Anti‐CD3 antibody (clone 17.A2, BD Biosciences) was added 10 min before the end of incubation. For the analysis of intracellular calcium, cells were treated in the same manner, with the exception of dye loading, which was performed using 1 μmol/L Indo‐1 (BD Biosciences) for 30 min at 37°C. Following incubation, cells were washed and prepared for flow cytometry analysis. Baseline fluorescence was recorded for 1 min, immediately followed by the addition of CCL‐19 (ROS assay: 40 nmol/L; Ca^2+^ assay: 40 and 400 nmol/L) for a duration of 3 min.

### 2.6 Statistics

Statistical analyses were conducted using GraphPad Prism 10 (GraphPad Software Inc). Data are shown as bar graph or dot plot with mean ± SD. Gaussian distribution was analyzed by Kolmogorov–Smirnov test, followed by Student's *t*‐test or Wilcoxon–Mann–Whitney test accordingly. *p*‐values < 0.05 were considered significant. If data are presented as normalized mean fluorescence intensity (MFI), in each experiment, the respective positive control was set to one, and all other depicted conditions were divided by the MFI of the positive control within each experiment. This ratio was created to exclude any influence of the different absolute values due to FACS or calibration, and therefore no standard deviation for the controls is presented. After normalization within each experiment, the average of three to four independent experiments was calculated and is depicted. In each experiment, appropriate solvent and negative controls were included but are not depicted for clarity of presentation purposes.

## 3 RESULTS

### 3.1 The murine LC‐AA allows for specific quantification of β2‐integrin–mediated affinity and avidity of T cells

**FIGURE 1:**
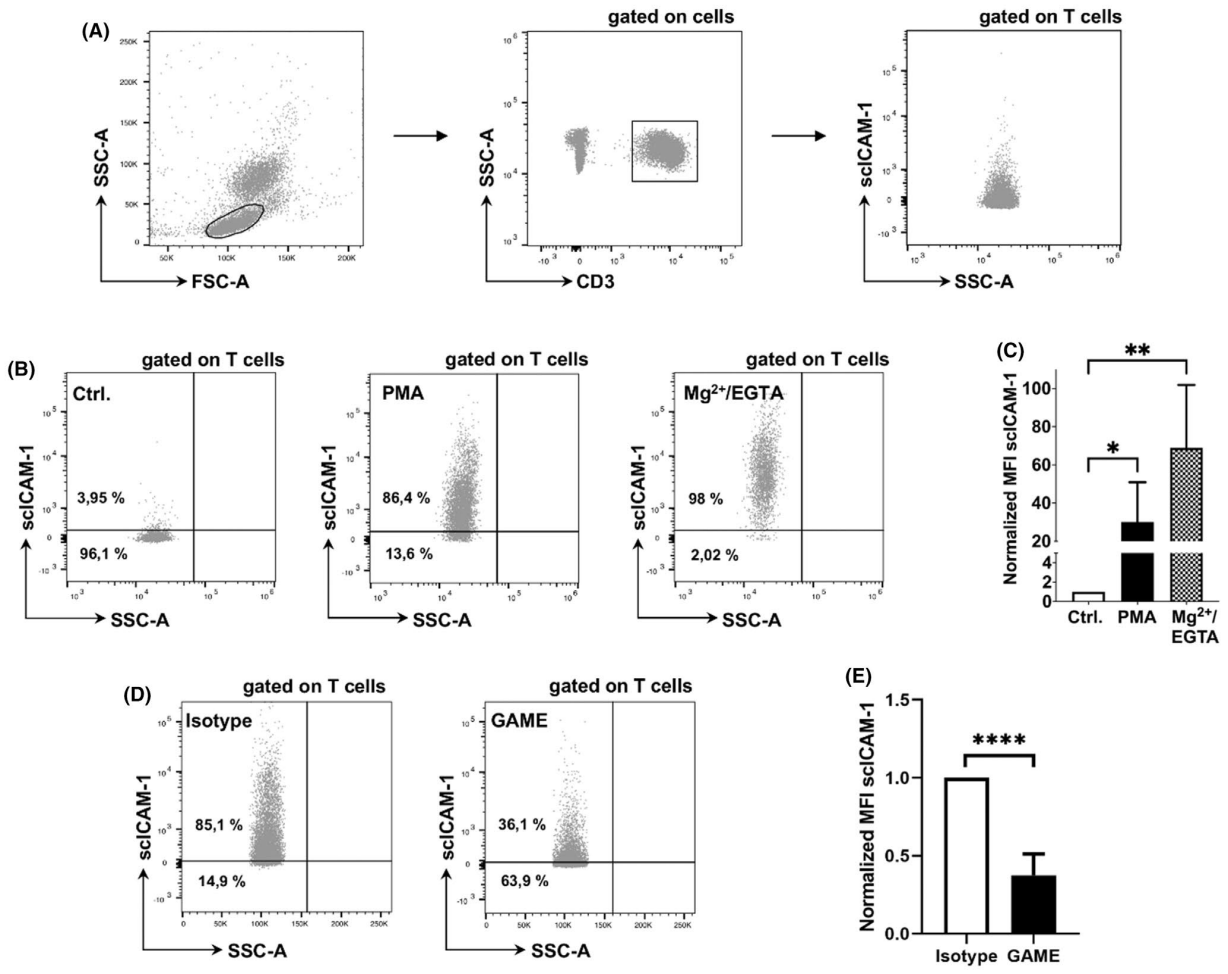
The murine LC‐AA allows specific quantification of β2‐integrin–mediated affinity and avidity of T‐cells in whole blood. (A) Gating strategy for flow cytometry: LC‐AA (right plot) was conducted after gating on T cells, which were defined as CD3^+^ cells (middle plot) after excluding cell debris and duplets by forward and side scatter plotting (left plot). (B) FACS analysis of murine whole blood samples, gated on T cells. The graphs show dot plots before (left graph) and after stimulation with PMA and Mg^2+/^EGTA. The number below the horizontal line depicts the percentage of T cells that were defined as unstimulated, whereas the percentage above indicates stimulated T cells. Stimulations with PMA and Mg^2+/^EGTA cause an increase in the percentage of stimulated cells. (C) The graph shows the normalized MFI of T cells of four independent experiments (*n* = 4 for each of the 3 groups) as well as respective standard deviation. In each experiment, the MFI values were normalized to the values of an unstimulated sample which was set as 100%. Stimulation with PMA leads to 30‐fold and Mg^2+/^EGTA to 69‐fold increase in scICAM‐1 MFI. (D) scICAM‐1 binding to murine T‐cells is integrin specific. After preincubation of murine whole blood leukocytes with blocking antibody (GAME: CD18 specific) and the respective IgG1 isotype control (clone R3–34) the LC‐AA was performed. The left representative dot plot shows PMA‐stimulated T cells after incubation with isotype control, the right dot plot after incubation with blocking antibody. (E) Incubation with CD18 blocking antibody results in a significant 63% decrease of scICAM‐1 MFI. The graph shows MFI values of five independent experiments (*n* = 5 for each of the two groups) after normalization to the mean fluorescence intensity of the respective isotype control. Data are shown as mean ± SD. *p ≤ 0.05, **p ≤ 0.01, ****p ≤ 0.0001.

To investigate β2‐integrin–mediated affinity and avidity of T cells by flow cytometry, we established a gating strategy as illustrated in Figure 1A. Cell debris and duplets were excluded by forward and side scatter plotting (left plot). Next, T cells were defined as CD3^+^ cells (middle plot), and mLC‐AA was carried out in this population (right plot). PMA is a prototypic stimulus for the induction of avidity, whereas Mg^2+/^EGTA is specific for the induction of integrin affinity. Incubation of whole blood leukocytes with PMA and Mg^2+/^EGTA causes an increase in the percentage of stimulated T cells (Figure 1B). Quantification of MFI reveals that stimulation with PMA leads to 30‐fold (*p* = 0.0316) and Mg^2+/^EGTA to 69‐fold (*p* = 0.0062) increase in scICAM‐1 MFI of murine T cells (Figure 1C). Murine whole blood leukocytes were incubated with an anti‐β2‐integrin antibody (GAME), and the respective IgG1 isotype control (Isotype) before mLC‐AA was conducted. Incubation with anti‐β2‐integrin antibody results in a significant 63% decrease (*p* < 0.0001) of scICAM‐1 MFI (Figure 1D,E). With these results, we demonstrate that the mLC‐AA allows specific quantification of β2‐integrin–mediated affinity and avidity, and that the assay is β2‐integrin specific (and blockable).

### 3.2 Naive but not memory T helper cells show strong increment of β2‐integrin–mediated adhesiveness after CCL‐19 stimulation

**FIGURE 2:**
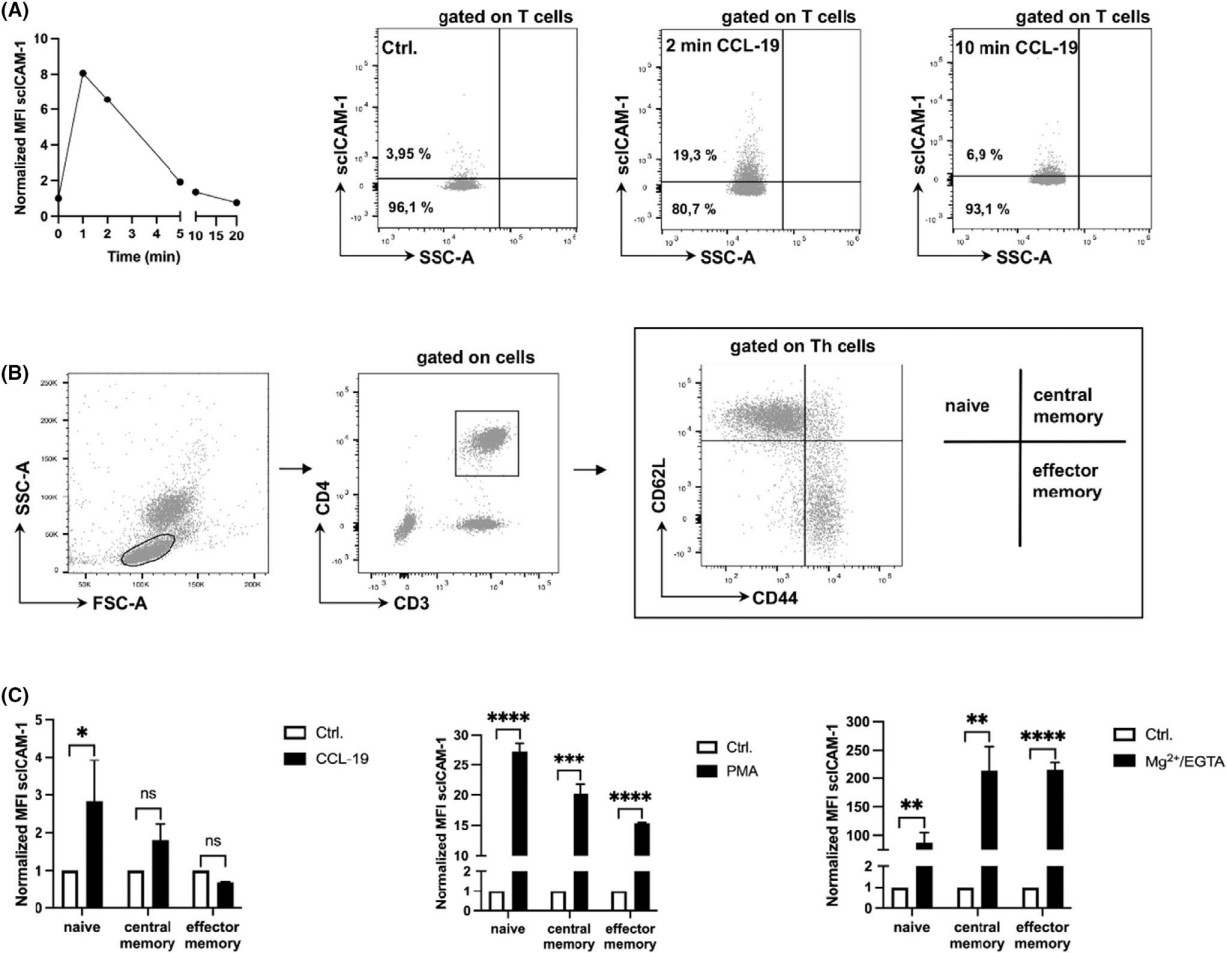
Naive but not memory T helper cells show strong increment of β2‐integrin–mediated adhesiveness after CCL‐19 stimulation. (A) FACS analysis of murine whole blood samples, gated on T cells. The graph on the left shows normalized scICAM‐1 MFI after CCL‐19 stimulation (ordinate) over the time in minutes (abscissa). The three representative dot plots on the right show scICAM‐1 of T cells before and after 2‐ or 10‐min stimulation with CCL‐19. (B) Gating strategy for flow cytometry: After excluding cell debris and duplets by forward and side scatter plotting (left plot) Th cells were defined as CD3^+^ CD4^+^ cells (middle plot), which were divided into naive, central, and effector memory Th cells based on their CD62L and CD44 expression. LC‐AA was then performed by gating on the respective Th cell subpopulation. (C) The graphs show the normalized MFI of scICAM‐1 of at least three independent experiments (*n* = 3 for each of the six subgroups of every graph). Stimulation with CCL‐19 (left graph) leads to a significant increase in normalized scICAM‐1 MFI in naive but not memory Th cells, whereas stimulation with PMA and Mg^2+/^EGTA leads to strong and significant increases in normalized scICAM‐1 MFI in all Th cell subsets. Data are shown as mean ± SD. ns *p* > 0.05, *p ≤ 0.05, **p ≤ 0.01, ***p ≤ 0.001, ****p ≤ 0.0001.

For the modulation of in vivo β2‐integrin adhesion in mice, the cytokine CCL‐19 was selected because it increases both integrin affinity and avidity, and therefore reflects a more physiological stimulus.^9^, ^11^ The mLC‐AA was carried out after stimulation of murine whole blood samples with CCL‐19 or a respective control. Normalized scICAM‐1 MFI in T cells peaks after 1–2 min before MFIs return to their initial unstimulated value around 10 min after stimulation (Figure 2A). To analyze subpopulations of T helper (Th) cells, a flow cytometric gating strategy to divide them into naive, central, and effector memory Th cells based on their CD62L and CD44 expression was established (Figure 2B). Stimulation with CCL‐19 (left graph) leads to a significant increase (*p* = 0.0485) in normalized scICAM‐1 MFI in naive but not in memory Th cells, whereas stimulation with PMA and Mg^2+/^EGTA leads to strong and significant increases in normalized scICAM‐1 MFI in all Th cell subsets (Figure 2C). Naive but not memory T helper cells show strong increment of β2‐integrin–mediated adhesiveness after CCL‐19 stimulation (Figure 2C).

### 3.3 Murine T cells show increased intracellular Ca^2+^ consumption and ROS production upon stimulation with CCL‐19

**FIGURE 3:**
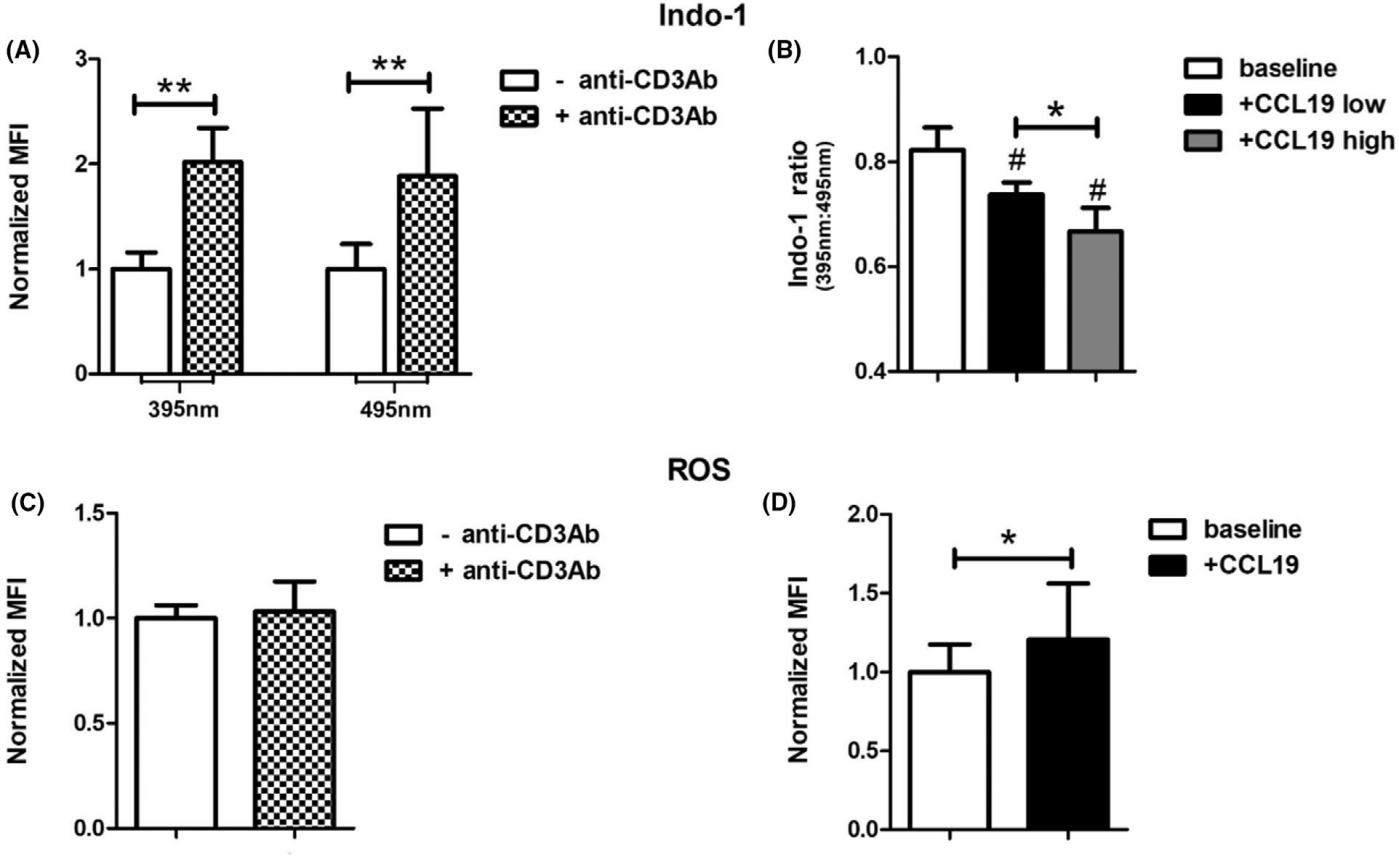
Murine T cells show increased intracellular Ca^2+^ consumption and ROS production upon stimulation with CCL‐19. (A) Shows the normalized MFI of the dye Indo‐1 at 395 nm (left graphs, bound Ca^2+^) and 495 nm (right graphs, free Ca^2+^), which both show a significant increase upon anti‐CD3‐antibody stimulation (*n* = 4). (B) Shows the Indo‐1 ratio (MFI of Indo‐1 at 395 nm/MFI of Indo‐1 at 495 nm) upon low‐ and high‐concentration CCL‐19 stimulation. Incubation with CCL‐19 (low: 40 nmol/L; high: 400 nmol/L) leads to a reduction in the Indo‐1 ratio, indicating the involvement of Ca^2+^ in chemokine‐mediated signaling (*n* = 6). (C) Shows the normalized MFI of the dye ROS Assay Stain Concentrate at 520 nm with no significant difference under anti‐CD3‐antibody stimulation (*n* = 4). (D) Shows the normalized MFI of the ROS Assay Stain Concentrate at 520 nm upon CCL‐19 stimulation, with significant higher MFI after CCL‐19 stimulation when compared to baseline control (*n* = 8). Data are shown as mean ± SD. *p ≤ 0.05, **p ≤ 0.01, ^#^ *p* ≤ 0.05 compared to baseline.

Ca^2+^ and ROS signaling is essential for β2‐integrin activation on human T cells upon chemokine stimulation.^19^ To test whether these signaling pathways also apply to murine β2‐integrin activation, we investigated direct Ca^2+^ and ROS production in T cells. Because T cell receptor (TCR) activation via CD3 already leads to intracellular accumulation of Ca^2+^,^23^, ^24^ we performed Ca^2+^ assays in the presence and absence of the anti‐CD3 antibody. Incubation with the antibody results in a significant (*p* = 0.0095) accumulation of intracellular free and bound Ca^2+^ (Figure 3A). Additional incubation with CCL‐19 leads to a dose‐dependent reduction in the Indo‐1 ratio (baseline vs. CCL low [*p* ≤ 0.01], baseline vs. CCL high [*p* ≤ 0.001], and CCL low vs. CCL high [*p* ≤ 0.05]), indicating the involvement of Ca^2+^ in chemokine‐mediated signaling (Figure 3B). After excluding the influence of the anti‐CD3 antibody on ROS production in these cells (Figure 3C), we performed experiments upon chemokine stimulation with CCL‐19 and found a significant increase (*p* = 0.0039) in ROS production compared to stimulation with solvent control (Figure 3D).

### 3.4 Enhanced β2‐integrin adhesiveness in T cells is ROS and Ca^2+^ dependent

**FIGURE 4:**
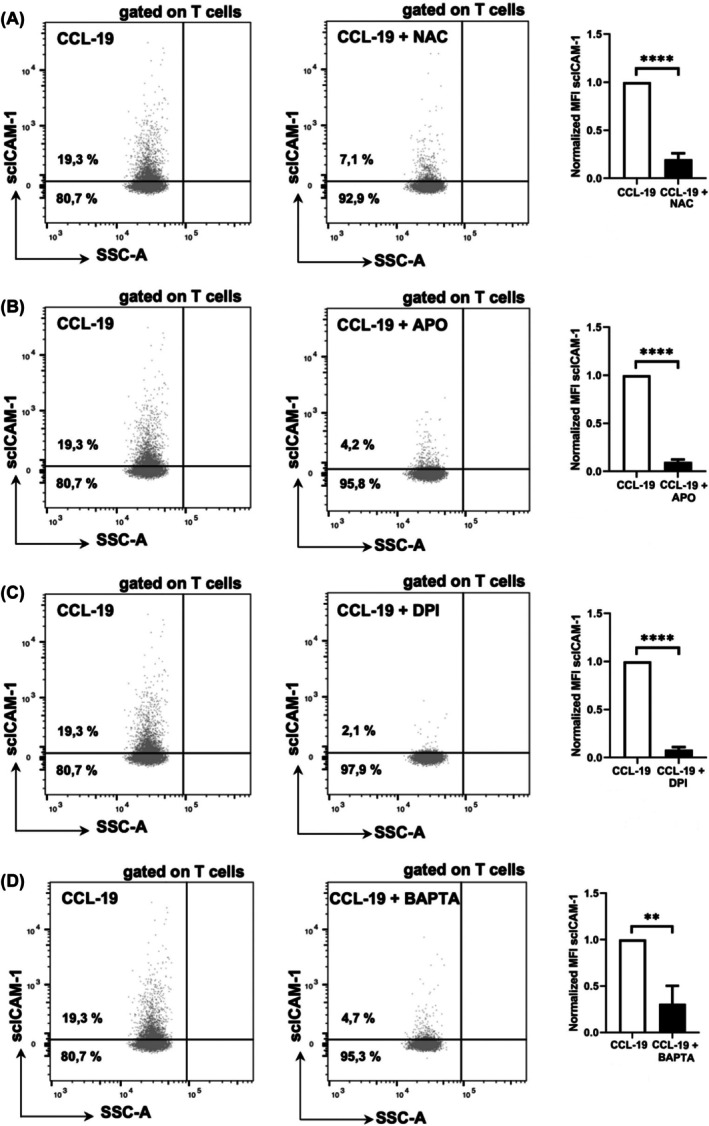
Enhanced β2‐integrin adhesiveness in T cells is ROS and Ca^2+^ dependent. FACS analyses of murine whole blood samples, gated on T cells. The dot plots show scICAM‐1 MFI after CCL‐19 stimulation without (PBS^+^ as solvent control, left graph) and with preincubation for 1 h with the reducing agents or calcium chelator. The number below the horizontal line depicts the percentage of T cells that were defined as unstimulated, whereas the percentage above indicates stimulated T cells. The bar graph on the right shows the MFI values of three independent experiments (*n* = 3) after normalization to CCL‐19 stimulation without inhibiting preincubation (*n* = 3). (A) Preincubation of scICAM‐1 with the reducing substance NAC (10 mmol/L) leads to a significant 80% decrease in scICAM‐1 MFI. (B) Preincubation of scICAM‐1 with the reducing substance APO (5 mmol/L) leads to a significant 90% decrease in scICAM‐1 MFI. (C) Preincubation of scICAM‐1 with the reducing substance DPI (20 μmol/L) leads to a significant 92% decrease in scICAM‐1 MFI. (D) Preincubation of scICAM‐1 with the calcium chelator BAPTA (200 μmol/L) leads to a significant 69% decrease in scICAM‐1 MFI. Data are shown as mean ± SD. **p ≤ 0.01, ****p ≤ 0.0001.

For functional testing of ROS and Ca^2+^ signaling in our assay, whole blood leukocytes were incubated with various reducing agents, and a calcium chelator before mLC‐AA was carried out. Dot plots in Figure 4 show scICAM‐1 MFI after CCL‐19 stimulation without and with preincubation with the aforementioned substances. Preincubation of scICAM‐1 with the reducing substances NAC, APO, and DPI for 1 hour leads to significant 80%, 90%, and 92% decreases (all *p* < 0.0001) of scICAM‐1 MFI (Figure 4A–C). Preincubation of scICAM‐1 with the calcium chelator BAPTA leads to a significant 69% decrease (*p* = 0.0035) of scICAM‐1 MFI (Figure 4D). These data indicate that β2‐integrin adhesiveness in murine T cells is ROS and Ca^2+^ dependent. The reducing agents showed no toxicity at the concentrations used; they rather have a protective effect on cell viability, probably due to their antioxidant properties (Figure S1).

### 3.5 Peripheral blood T cells of mice suffering from MI exhibit elevated β2‐integrin adhesion capacity

**FIGURE 5:**
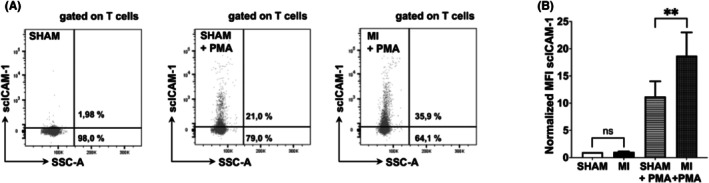
T cells of mice suffering from myocardial infarction exhibit elevated β_2_‐integrin adhesion capacity. (A) The left representative dot plot shows scICAM‐1 of unstimulated (PBS^+^ as solvent control) whole blood leukocytes of a sham‐operated mouse (SHAM), the middle representative dot plot shows scICAM‐1 of PMA‐stimulated whole blood leukocytes of a sham‐operated mouse (SHAM+PMA), and the right dot plot shows scICAM‐1 of PMA‐stimulated whole blood leukocytes of a mouse that underwent myocardial infarction (MI + PMA) by permanent LAD ligature. The number below the horizontal line depicts the percentage of T cells that were defined as unstimulated, whereas the percentage above indicates stimulated T cells. LC‐AA of whole blood leukocytes of mice after MI shows an increase in the percentage of stimulated T cells. (B) Whole blood leukocytes of mice with MI (*n* = 6) show no difference in scICAM‐1 MFI compared to mice with SHAM (*n* = 6). Upon stimulation with PMA, whole blood leukocytes of mice with MI (*n* = 6) show 85% increase in scICAM‐1 MFI compared to mice with SHAM MI (*n* = 6). The graph shows MFI values of six independent experiments after normalization to the mean fluorescence intensity of the respective sham animals (SHAM). Data are shown as mean ± SD. ns, *p* > 0.05; **p ≤ 0.01.

In patients with MI, the extent of β2‐integrin activation of T cells correlates with future cardiovascular events.^21^ To test whether a similar association takes place in mice, MI was induced by permanent LAD ligation, and whole blood samples were harvested after 7 days by intracardial punctation. Representative dot plots show scICAM‐1 of unstimulated whole blood leukocytes of a sham‐operated mouse (SHAM), PMA‐stimulated whole blood leukocytes of a sham‐operated mouse (SHAM+PMA) and PMA‐stimulated whole blood leukocytes of a mouse that underwent myocardial infarction (MI + PMA) by permanent LAD ligature. Spontaneous scICAM‐1 binding of T cells in MI/SHAM mice was comparable to those of mice without interventions and 30× lower than after PMA stimulation (see Figure 1). We did not detect a difference in scICAM‐1 MFI between SHAM and MI mice under unstimulated conditions. After stimulation of whole blood samples in vitro for 30 min with PMA, T cells of mice after MI show an increase in the percentage of stimulated T cells (Figure 5A). Unstimulated (PBS^+^ was used as solvent control) whole blood samples of sham‐operated mice served as additional controls (Figure 5A). PMA‐stimulated whole blood samples of mice with MI show an 85% increase in scICAM‐1 MFI compared to mice with SHAM (*p* = 0.0064) (Figure 5B). We conclude that MI causes β2‐integrin activation of T cells in mice after PMA stimulation.

## 4 DISCUSSION

Integrin adhesion is pivotal for leukocyte extravasation in inflammatory diseases. Integrin‐β2 controls the adhesion of leukocytes via binding of ICAM‐1.^5^ Lack of integrin‐β2 causes leukocyte adhesion deficiency, resulting in severe immunodeficiency with increased susceptibility for infections.^25^ Importantly, integrins while highly expressed on the cell surface are not functional until activated.^2^, ^8^ The complex activation process is mediated by changes in receptor affinity or avidity,^6^ which can be analyzed in human cells^13^ and whole blood^14^ by applying the ligand‐complex–based adhesion assay (LC‐AA). Assays for the analysis of integrin adhesiveness have been developed primarily for human cells.^13^, ^14^ They are unsuitable for preclinical animal models that limit research in this area. To address this problem, the murine ligand‐complex–based adhesion assay (mLC‐AA) for the specific quantification of integrin adhesiveness in mice is presented. The mLC‐AA is suitable for the analysis of β2‐integrin activation on murine T cells in whole blood samples, which was confirmed by incubation with PMA and Mg^2+/^EGTA. This makes the assay suitable for the detection of both avidity and affinity changes of integrin‐β2 in mice without prior cell purification. Specificity of the mLC‐AA was verified by preincubation with an anti‐β2‐integrin antibody (anti‐CD18–specific IgG [clone GAME]), which resulted in a significant decrease in ICAM‐1 binding to T cells when compared to isotype control. However, blockade of the antibody is still incomplete. This could be due to the relatively high expression of CD18, which leads to only incomplete saturation. The assay allows the measurement of very small volumes of blood (20 μL, e.g., by puncture of the facial vein) making repetitive measurements possible without the need to kill the animal. This is particularly important when investigating valuable transgenic animals.

Despite prognostic and interventional improvements, CCS and its most severe clinical presentation MI remain the leading causes of death worldwide.^26^ T cells are recruited to the atherosclerotic plaque in a β2‐integrin–dependent manner and contribute to plaque destabilization.^19^, ^20^, ^21^ Assessment of β2‐integrin activation on T cells in patients with ACS correlates with the risk for future cardiovascular events.^21^ In line with that, we could show that β2‐integrin adhesion capacity of murine T cells is elevated after MI upon PMA stimulation but not under basal conditions. The lack of difference under unstimulated conditions is not consistent with previous work, where patients suffering from MI showed significantly higher scICAM‐1 MFI levels when compared to controls.^21^ However, this resistance in β2‐integrin adhesion capacity may be a possible explanation why mice are more resistant to plaque rupture than human beings.^27^, ^28^ The inflammatory aspect in the pathogenesis of atherosclerosis has been well understood in recent years.^15^, ^16^ Clinical trials with cytokine‐targeting antibodies^29^ or colchicine^30^ have been presented without uncovering specific molecular targets, implicating that there is a further need of preclinical research models to tackle inflammation in patients suffering from atherosclerosis.

Recently, it was shown that ROS through hydrogen peroxide modulate β2‐integrin activation after chemokine stimulation of human T cells by Ca^2+^ release.^19^ In congruence, we demonstrate that chemokine stimulation of murine T cells with CCL‐19 increases ROS production. TCR activation via CD3 or CD4 leads to intracellular accumulation of Ca^2+^.^23^, ^24^ We show that binding of anti‐CD3 antibody to T cells with consecutive TCR activation releases Ca^2+^. Probably due to this significantly increased intracellular accumulation of Ca^2+^, there appears to be no further CCL19‐dependent release. However, we present a dose‐dependent reduction in the Indo‐1 ratio, indicating an intracellular consumption of Ca^2+^ upon CCL‐19 stimulation. By applying the mLC‐AA assay, we were able to demonstrate that murine signaling of β2‐integrin adhesion upon chemokine stimulation is ROS‐ and Ca^2+^‐dependent. These findings imply that murine and human β2‐integrin signaling shares similar molecular mechanisms, making the mLC‐AA a valuable tool for translational research. The mLC‐AA enables functional analyses, as the target populations are only stained after fixation, thus preventing the antibodies from interfering with underlying ROS and Ca^2+^ signaling.

Fixation of cells with formaldehyde immediately terminates the adhesion process, which makes the mLC‐AA suitable for time course analyses. This could be demonstrated by different incubation times with CCL‐19 to find the time point when maximal adhesiveness was reached. Utilization of low whole blood volumes without preliminary sample processing allows analysis of functional T cell behavior as closely as possible to the in vivo situation. In addition, the whole blood approach opens up the opportunity of analyzing heterogeneous T cell subpopulations. Analysis of Th cell subsets revealed that stimulation with CCL‐19, a potent inductor of T cell activation,^31^ increases β2‐integrin adhesion in naive but not in memory Th cells. This can be explained by a higher expression of CCR7, the CCL‐19 receptor, in naive Th cells compared to memory or effector Th cells.^32^ On the contrary, naive Th cells express only low levels of CD18 because they primarily recirculate through secondary lymphoid organs using low‐affinity interactions, whereas memory and effector Th cells require higher CD18 levels to facilitate rapid adhesion to inflamed tissues.^33^, ^34^ Increased scICAM‐1 MFI in naive but not in memory Th cells can therefore not be explained by higher CD18 expression in this subset.

In conclusion, the mLC‐AA allows specific quantification of cellular adhesiveness as a consequence of changes in β2‐integrin affinity and avidity at the single cell level of murine T cells. We were able to show that ROS/Ca^2+^ signaling is essential for murine β2‐integrin activation upon chemokine stimulation. Functional analysis of integrin activity may represent a novel tool for the characterization of signaling cascades based on the functional behavior of ex vivo cells. Furthermore, it may be possible to identify particularly reactive subpopulations, which contribute to inflammatory diseases such as CCS or MI.

## AUTHOR CONTRIBUTIONS

**M. Therre:** Conceptualization; data curation; formal analysis; investigation; project administration; visualization; writing – original draft. **A. A. Kuhnle:** Data curation; formal analysis; investigation. **H. M. Arndt:** Data curation; formal analysis; investigation. **N. Frey:** Conceptualization; funding acquisition; resources; software; supervision; writing – review and editing. **M. H. Konstandin:** Conceptualization; formal analysis; funding acquisition; methodology; project administration; resources; software; supervision; writing – review and editing. **N. V. Bogert:** Conceptualization; data curation; formal analysis; funding acquisition; investigation; methodology; project administration; resources; software; supervision; validation; writing – review and editing.

## FUNDING INFORMATION

This work was supported by the DZHK (German Centre for Cardiovascular Research).

## CONFLICT OF INTEREST STATEMENT

The authors declare no conflicts of interest regarding this manuscript.

## ETHICS STATEMENT

All conducted animal experiments of this study were reviewed and approved by the Regierungspräsidium Karlsruhe (G246/14; T24/19).

## Supporting information


**Figure S1.** The reducing agents DPI, APO, and NAC are not toxic at the used concentrations. (A) FACS analyses of murine T cells. The dot plots show the proportion of dead cells (FVS+), the larger FVS‐ population are living cells. The plot on the left shows PBS^+^ as solvent control (Ctrl.), with 8.29% dead cells, while preincubation with the reducing substance DPI (20 μM) leads to a proportion of 2.34% dead cells, with APO (5 mM) to a proportion of 3.41% dead cells and with NAC (10 mM) to a proportion of 6.76% dead cells, respectively.
