# Supplementary material for: Ligand‐complex–based quantification of β2‐integrin–mediated affinity and avidity of murine T cells
Source: Animal Model Exp Med. 2026 Feb 25;9(2):319–28. doi: 10.1002/ame2.70155 (PMC13042816; doi:10.1002/ame2.70155)
Supplement: Supplementary file 1 — Figure S1. The reducing agents DPI, APO, and NAC are not toxic at the used concentrations. (A) FACS analyses of murine T cells. The dot plots show the proportion of dead cells (FVS+), the larger FVS‐ population are living cells. The plot on the left shows PBS+ as solvent control (Ctrl.), with 8.29% dead cells, while preincubation with the reducing substance DPI (20 μM) leads to a proportion of 2.34% dead cells, with APO (5 mM) to a proportion of 3.41% dead cells and with NAC (10 mM) to a proportion of 6.76% dead cells, respectively. [file AME2-9-319-s001.docx]

**Supplemental Figure 1**

**Supplemental Figure 1: The reducing agents DPI, APO, and NAC are not toxic at the used concentrations. (A)** FACS analyses of murine T cells. The dot plots show the proportion of dead cells (FVS+), the larger FVS- population are living cells. The plot on the left shows PBS^+^ as solvent control (Ctrl.), with 8,29% dead cells, while preincubation with the reducing substance DPI (20 µM) leads to a proportion of 2,34% dead cells, with APO (5 mM) to a proportion of 3,41% dead cells and with NAC (10 mM) to a proportion of 6,76% dead cells, respectively.
